# Supplementary material for: Identification of Quantitative Trait Locus and Candidate Genes for Drought Tolerance in a Soybean Recombinant Inbred Line Population
Source: Int J Mol Sci. 2022 Sep 16;23(18):10828. doi: 10.3390/ijms231810828 (PMC9504156; doi:10.3390/ijms231810828)
Supplement: Supplementary file 1 [file ijms-23-10828-s001.zip › ijms-1846731-supplementary.pdf]

Supporting Information

Identification of Quantitative Trait Locus and candidate genes for drought tolerance in a soybean Recombinant Inbred Line population

Wenqi Ouyang[1#] Limiao Chen[1#] Junkui Ma[2], Xiaorong Liu[2], Haifeng Chen[1], Hongli Yang[1], Wei Guo[1], Zhihui Shan[1], Zhonglu Yang[1], Shuilian Chen[1], Yong Zhan[3], Hengbin Zhang[3], Dong Cao[1*], and Xinan Zhou[1*].

[1]Key laboratory of Biology and Genetic Improvement of Oil Crops, Ministry of Agriculture and Rural Affairs, Oil Crops Research Institute of Chinese Academy of Agricultural Sciences, Wuhan, China 430062

[2]The Industrial Crop Institute, Shanxi Academy of Agricultural Sciences, Taiyuan, China 030006

[3]Crop Research Institute, Xinjiang Academy of Agricultural and Reclamation Science, Key Lab of Cereal Quality Research and Genetic Improvement, Xinjiang Production and Construction Crops, Shihezi, China 832000

*Corresponding authors:

Xin-An Zhou,

Chinese Academy of Agriculture Sciences, Oil Crops Research Institute, Xudong 2nd Road, Wuhan, China 430062, Phone: 86-027-86711563, FAX: 86-027-86711563, Email: zhouocri@sina.com

Dong Cao,

Chinese Academy of Agriculture Sciences, Oil Crops Research Institute, Xudong 2nd Road, Wuhan, China 430062, Phone: 86-027-86711563, FAX: 86-027-86711563, Email: caodong@caas.cn;

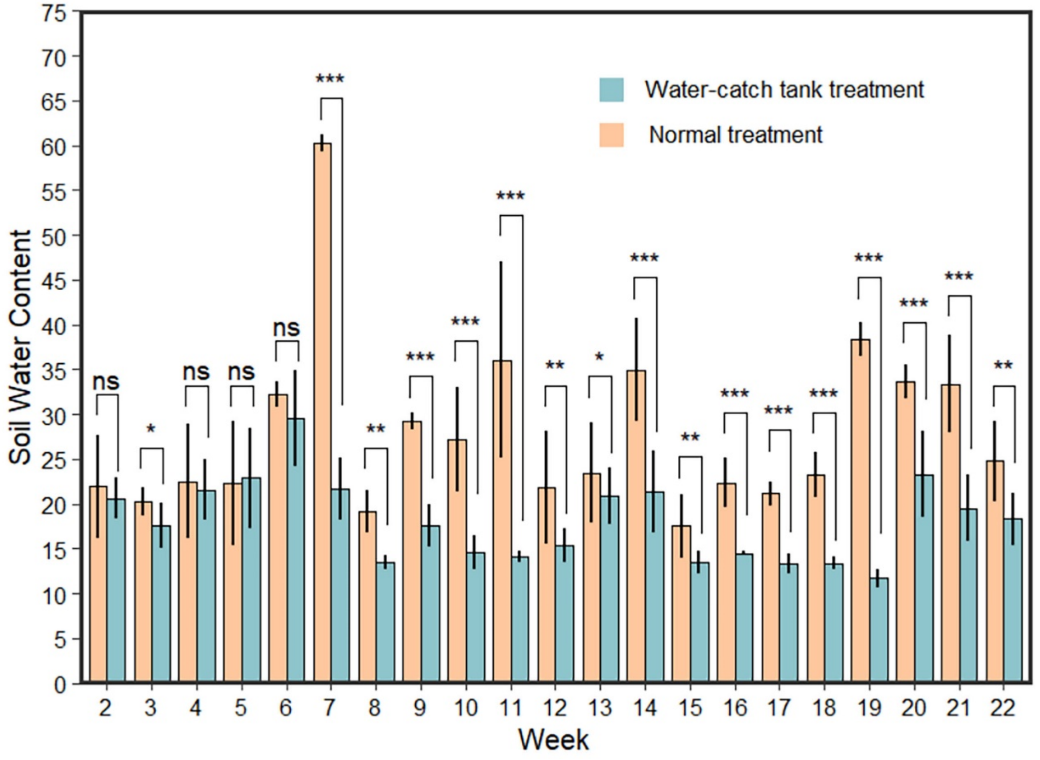

Supplementary Figure S1. Comparison of soil water content between normal and catchment tank treatments over 22 weeks. X-axis represents week of drought treatment, and y-axis represents soil water content (%).Statistically significant differences of soil water content between normal treatment and drought treatment (water-catch tank treatment) are marked with asterisks (*P < 0.05, **P<0.01 and *** P < 0.001, Student's t-test. ns, non-significant).

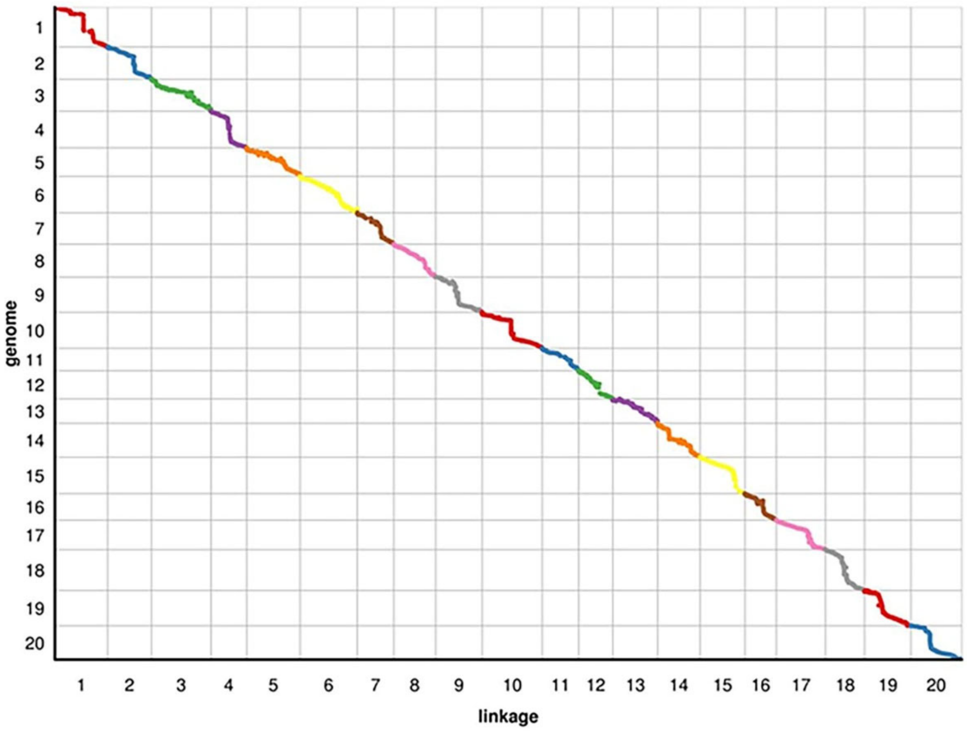

Supplementary Figure S2. Relationship between genetic and physical positions with each chromosome. In each plot, genetic distance is on the x-axis, and physical distance is on the y-axis.
